# Comparing wMAS, GWAS, and genomic prediction for selecting powdery mildew-resistant spring barley genotypes

**DOI:** 10.1186/s12864-025-12395-y

**Published:** 2025-12-05

**Authors:** Su Myat Noe, Pawan Kumar Singh, Firuz Odilbekov, Eva Johansson, Aakash Chawade

**Affiliations:** 1 Department of Plant Breeding, Swedish University of Agricultural Sciences, Alnarp, Sweden; 2 International Maize and Wheat Improvement Center (CIMMYT), Veracruz, El Batan, Texcoco, 56237 Mexico; 3 Lantmännen Lantbruk, Svalöv, Sweden

**Keywords:** GWAS, wMAS; genomic prediction, Barley, Powdery mildew, rrBLUP

## Abstract

**Background:**

Barley is one of the most widely cultivated cereals worldwide, and powdery mildew is among the major diseases threatening global barley production. Our study evaluated 370 spring barley breeding lines under controlled greenhouse growth conditions.

**Results:**

Using genome-wide association study (GWAS), 21 quantitative trait loci (QTL) were identified associated with seedling-stage powdery mildew resistance. Of these, eight were newly identified in this study. Genetic merit was also calculated using major-effect markers, and a positive correlation (> 0.7) was observed between the genetic merit and BLUP (AUDPC) values in both the two subpopulations of two- and six-row barley. While evaluating the performance of genomic prediction (GP) models, a GWAS-incorporated GP model consistently outperformed the Standard GP model in both subpopulations demonstrating the advantage of incorporating major-effect markers for a more accurate prediction. Our analysis of genotype selection patterns revealed a notable degree of agreement among the tested methods. In the two-row subpopulation, a large number of genotypes were exclusively selected by weighted marker-assisted selection (wMAS) revealing the dominance of major-effect QTL. In contrast, the six-row subpopulation had a smaller wMAS-exclusive group, suggesting a more polygenic background, which was captured by genomic prediction. Additionally, genomics-based methods consistently identified resistant genotypes that were overlooked by phenotypic selection, showing their ability to detect hidden genetic potential.

**Conclusions:**

Overall, GWAS-incorporated GP model demonstrated the best performance among the evaluated methods, suggesting this approach is the most effective with a potential to contribute to efficient breeding of powdery mildew resistance in spring barley.

**Supplementary Information:**

The online version contains supplementary material available at 10.1186/s12864-025-12395-y.

## Background

Barley (*Hordeum vulgare* L.) is a domesticated crop with a long history originating in the Fertile Crescent [1]. Due to its resilience to unfavorable climate conditions such as drought and poor soil fertility, it is considered an important cereal crop [2]. Barley is the fourth-largest crop globally, with an estimated production of 145 million tons in 2023.

Powdery mildew (PM), caused by the biotrophic fungus *Blumeria graminis* f. sp. *Hordei (Bgh)*, is a major disease on barley affecting production worldwide, although in particular in temperate regions. *Bgh* causes white patches on the barley leaves and stems, which disturb photosynthesis and reduce yield [3]. Disease management in barley production relies on both fungicides and resistant varieties, though genetic resistance remains the most sustainable solution [4]. Traditionally, phenotypic evaluation has been the primary method to select superior genotypes, although such methodologies are time-consuming and often require several generations of the crop for a proper selection. The dawn of genomics tools facilitated the first QTL mapping through linkage analysis, even though these approaches heavily relied on pedigree along with phenotypic information [5].

More than 30 distinct PM resistance genes have been identified in barley, providing important information for breeding resistant cultivars [6, 7]. Major resistance genes such as *Mla1*,* Mla6*,* Mla12*,* Mlg*,* Mlp*,* Mlk*, and *Mlh* provide race-specific resistance to distinct *Bgh* isolates [8]. Another well-known PM-resistant gene is *mlo*, whose resistance is mediated by a recessive mechanism. Unlike the race-specific resistant genes (major genes) described above, *mlo* provides a broad-spectrum durable resistance and inhibits pathogen growth at the earliest stage of infection [6]. Recent developments such as genome-wide association study (GWAS) provides significant advantages over QTL mapping by using a greater allelic diversity [9]. However, both QTL mapping and association mapping are still useful tools for breeding programs by enabling the exploration of QTL associated with various traits.

Recently, genomic prediction (GP) has increased in popularity as this methodology is predicting the breeding values of individuals based on their genomic information without their phenotypic data [10]. A GP model is trained by using a specific training population with known genotypic and phenotypic data, and thereafter, the genomic estimated breeding values (GEBVs) of an independent population can potentially be calculated without prior phenotypic data. The predictive ability (PA) of the model is measured by correlating the predicted values with the observed values [11]. Many factors can influence the PA including the size of training population, quality of phenotypic data and trait complexity [10].

GP studies utilize models, including ridge regression best linear unbiased prediction (rrBLUP) and Bayesian models, both of which treat all markers as random effects [12]. Additionally, models combining random- and fixed-effects has also been employed [13]. Each of the models are employing a different algorithm, contributing to the challenges of determining which model is preferable to use [14, 15]. Thus, in a study aimed to predict seedling stage resistance against Septoria Tritici Blotch in mixed winter wheat cultivars, the GP model, incorporating GWAS-significant markers as fixed effects, resulted in more significant predictions than rrBLUP, which used all markers as random effects [16]. However, the rrBLUP model was superior to the GWAS-incorporated GP model for the predicting of spot blotch resistance in bread wheat breeding populations [13].

With the increasing application of genomic tools in plant breeding, a comprehensive and direct comparison of different genotype selection methods are becoming important. Such a comparison should include traditional phenotypic selection (PS), weighted marker-assisted selection (wMAS), and genomic selection (GS), since each of these methods have its own advantage for capturing the underlying genetic variation, which is then optimized in breeding strategies [17, 18]. Due to the significant effects of powdery mildew on barley, it is strategically important to identify the most effective and efficient selection strategies to improve resistance, and to make targeted decisions in practical plant breeding.

This study evaluated 370 advanced spring barley breeding lines developed by Lantmännen AB, Sweden, with the following aims: (1) to identify QTL associated with seedling stage powdery mildew resistance in spring barley using GWAS (2) to develop and evaluate GP models for powdery mildew resistance considering different training populations and marker sets (3) to evaluate four selection methods: PS, wMAS, and two GS strategies, i.e. a Standard GP approach, and a GWAS-incorporated GP approach for identifying superior breeding lines for powdery mildew resistance.

## Methods

### Plant materials and growth conditions

A total of 370 advanced spring barley breeding lines (219 lines for two-row and 151 lines for six-row type) were provided by Lantmännen and tested for powdery mildew resistance under controlled conditions at Svalöv, Sweden, in 2022 and 2023. Two commercial cultivars, Anneli (two-row cultivar) and Judit (six-row cultivar), were included as checks in both experiments. All these materials were randomly arranged in an Alpha Lattice design with three replicates and the whole experiment was repeated twice. Approximately ten seeds per genotype were grown with 10 × 10 cm spacing in the aluminum benches in the greenhouse. Seedlings were watered with a sprinkler once a day, and the light condition was maintained at a 16-hour light/8-hour dark cycle.

### Inoculation and disease evaluation

**Fig. 1:**
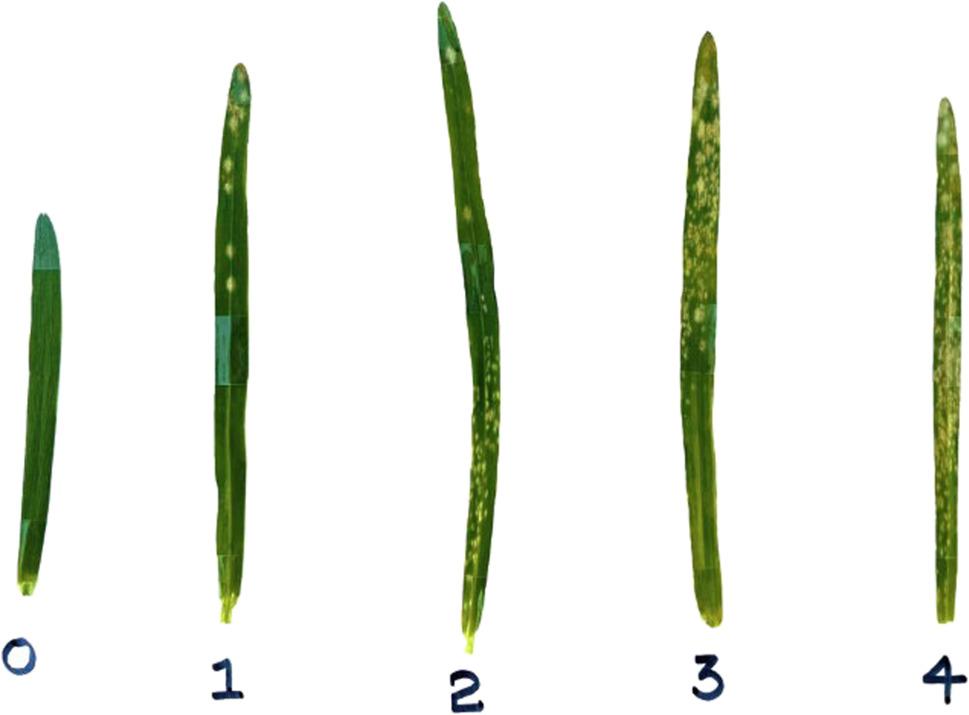
Powdery mildew disease scales ranging from 0 to 4 are used in current experiment. 0 represents the least infection (with almost no infection), and the infection increases as the number increases, reaching a maximum of almost 100% at score 4. Scoring was done by each genotype row by using the scoring categories in this figure. (Blue shading appeared on some leaves is due to the reflection of adhesive tape used during imaging and does not represent a biological feature.)

Powdery mildew-infected leaves were collected from the barley growing fields in Svalöv. Inoculation relied on field-collected powdery mildew samples, and the *Bgh* isolates present were not characterized. Being an obligate pathogen, *Bgh* was grown on live barley plants using the cultivars Anneli and Judit. One day before inoculation, the old conidia were removed by shaking them off from the cultured plants to get fresh and infectious inoculum. The inoculation was carried out twice, 9 days and 11 days after germination, by directly brushing infected plants against the uninfected test plants in both experiments. The growing chamber was maintained at constant light/dark and humidity conditions, as mentioned in the plant materials and growth conditions section. Disease scoring was performed three times (11, 13, and 15 days post inoculation, DPI) during the first experiment and four times (14, 16, 18, and 20 DPI) during the second experiment. This difference in scoring timing and frequency was adjusted based on the observed differences in disease progression rate between the two experiments. The disease severity was assessed at the whole-plot level by assigning a single mean score on a 0–4 scale (5 categories, of which 0 represents no infection and 4 represents the highest infection, ≈ 100% severity) [6]. (Fig. 1).

Disease scores collected at each time point were adjusted for the partially balanced incomplete design (PBIB) using PBIB.test function from the Agricolae package following the statistical model [19, 20].

$$\text{Y}_\text{ilm}=\mathrm\upmu+\text{G}_\text{ilm}+\text{B}_\text{lm}+\text{R}_\text{m}+\mathrm\upvarepsilon_\text{ilm}$$

where: Y_ilm_ is the score of i^th^ genotype in the l^th^ block at the m^th^ replicate; µ is the overall mean; G_ilm_ is the effect of i^th^ genotype in the l^th^ block at the m^th^ replicate; B_lm_ is the block effect at m^th^ replicate; R_m_ is the m^th^ replicate and ε_ilm_ is the residual error.

These adjusted means were used to calculate area under disease progress curve (AUDPC) [21].

$$\text{AUDPC}=\sum\nolimits_{\text{i}=1}^{\text{n}-1}\left(\frac{\text{y}_\text{i}+\text{y}_\text{i+1}}{2}\right)\left(\text{t}_{\text{i}+{\text1}}-\text{t}_\text{i}\right)$$

, where yi is the score at ith observation; ti is the time (DPI) at the ith observation and n is the number of observations (genotypes).

Finally, best linear unbiased prediction (BLUP) was calculated for each genotype using the AUDPC values from the two experiments with the following statistical model in META-R software [22].

$$\text{Y}_\text{il}=\upmu+\text{Gen}_\text{i}+\text{Rep}_\text{l}+\upvarepsilon_\text{il}$$

, where Yil is the AUDPC of the ith genotype in the lth replicate (experiment), µ is the overall mean, Gil is the ith genotype effect in the lth replicate (experiment) and εil is the residual effect.

### Marker information and quality control

The tested breeding materials were genotyped with 15 K Illumina Infinium array from TraitGenetics GmbH (SGS, Germany) (https://sgs-institut-fresenius.de/en/gesundheit-und-ernaehrung/traitgenetics). Marker information, including physical position, chromosome, and reference genome, was retrieved from the Triticeae Toolbox (T3) [23] and BARLEYMAP [24]. Markers with more than 10% missing data and those with minor allele frequency (MAF) of less than 5% were removed using TASSEL software version 5.2.93 [25]. After filtering, 8,088 SNPs for two-row subpopulation and 6,915 SNPs for six-row subpopulation remained for GWAS analysis.

### Genome-wide association study (GWAS)

GWAS was performed for two-row and six-row subpopulations separately to minimize the cofounding effects related to the population structure and maximize the identification of population-specific markers, avoiding the masking effect of a combined analysis [26–28], using BLINK [29] model from the GAPIT package (version 3.4) in R 4.3.3 [30]. Significantly associated markers were identified by setting the False Discovery Rate (FDR) at 0.05 and applying the Bonferroni threshold (0.05 divided by the number of filtered SNPs). The Bonferroni thresholds were 6.18 e^− 6^ and 7.23 e^− 6^ for two-row and six-row subpopulations, respectively.

To characterize the extent of linkage disequilibrium (LD) across each chromosome, pairwise LD (r^2^) between all SNP markers was calculated in TASSEL [25] with a sliding windows 50 SNPs [31]. The LD (r^2^) values were plotted against physical distance (Mbp) for each chromosome. Chromosome-wide LD decay plots are presented in Supplementary Figure S1 and S2. For QTL identification, marker-trait associations (MTAs) within the LD decay distance were considered to represent the same locus [32].

### Weighted marker-assisted selection within spring barley subpopulations

GWAS was performed for each subpopulation as described above, using 6,915 markers for the six-row and 8,088 markers for the two-row populations. For the two-row population, only one significant marker was detected for powdery mildew resistance; therefore, wMAS for this group relied on genotype classes at this single locus. The genetic contribution of a marker was estimated by multiplying the marker’s effect size (i.e., additive effect obtained from GWAS results) by the genotype’s allele composition (0 = no favorable allele, 1 = one favorable allele, or 2 = two favorable alleles). The cumulative genetic values were predicted by summing the contributions of individual markers according to the following equation [33].

$$\text{Y}=\upmu+\sum\nolimits_{i=1}^{n}{a}_{i}{b}_{i}+\upvarepsilon$$

Where: Y is the predicted genetic value; µ is the overall mean; $\:{a}_{i}$ is the estimated additive effect of the i^th^ marker; $\:{b}_{i}$ is the genotype’s allele composition at the i_th_ marker; ε is the residual.

Genotypes with the lowest predicted genetic values were considered most resistant, following the assumption that lower BLUP (AUDPC) values reflect reduced disease severity, as aligned with the wMAS selection criterion used in this study.

### Genomic prediction using standard and GWAS-incorporated models

Beyond the direct application of major QTL in wMAS, we also investigated the potential of genomic prediction to improve the breeding efficiency of powdery mildew resistance in spring barley. Using filtered genotypic data, GP analyses were primarily performed for each subpopulation using mixed.solve function from rrBLUP package (version 4.6.3), based on the following linear mixed model [34]:

$$\text{Y}=\text{X}\upbeta+\text{Z}\upmu+\upvarepsilon$$

where: Y is the vector of adjusted BLUP (AUDPC) values; X and β are the design matrix and vector of fixed effects; Z and µ are the design matrix and vector for random effects (i.e., filtered SNP markers which varied by population); ε is the residual.

Each tested population was randomly divided into a training set (80%) and a validation set (20%). Two main GP strategies were applied.

- *GWAS_FIX_rrBLUP*: This model included Bonferroni-significant markers identified via GWAS as fixed effects, while the remaining markers were treated as random effects. GWAS was performed within each training set independently. The significant markers from each run were then incorporated into the prediction model. This strategy was repeated 20 times, and PA was recorded for each run.
- *STD_rrBLUP*: In this model, no marker was preselected; all markers were included as random effects. This model was repeated 500 times, and PAs were calculated.

### Genotype selection: phenotypic, genotypic, and weighted marker-assisted selection

Afterward, we evaluated PS, GS, and wMAS to determine which genotypes were selected or discarded by one or more of the methods. Due to random splitting of the population, genotypes may appear multiple times in the validation sets. Therefore, the mean GEBV for each genotype was calculated by averaging across all its validation appearances. Based on the average GEBVs, two selection thresholds were defined: the top 10% and 20% of genotypes with the lowest disease incidence, i.e., the lowest BLUP for PS and the lowest GEBVs for GS, and the lowest genetic merit for wMAS. However, unlike other selection methods that used GEBVs and BLUPs to define top percentiles, wMAS in the two-row subpopulation relied on discrete marker scores (genetic merit) derived from a single significant GWAS marker, with only genotypes carrying the favorable allele being selected.

## Results

### Selection method 1: phenotypic evaluation of powdery mildew resistance

**Fig. 2:**
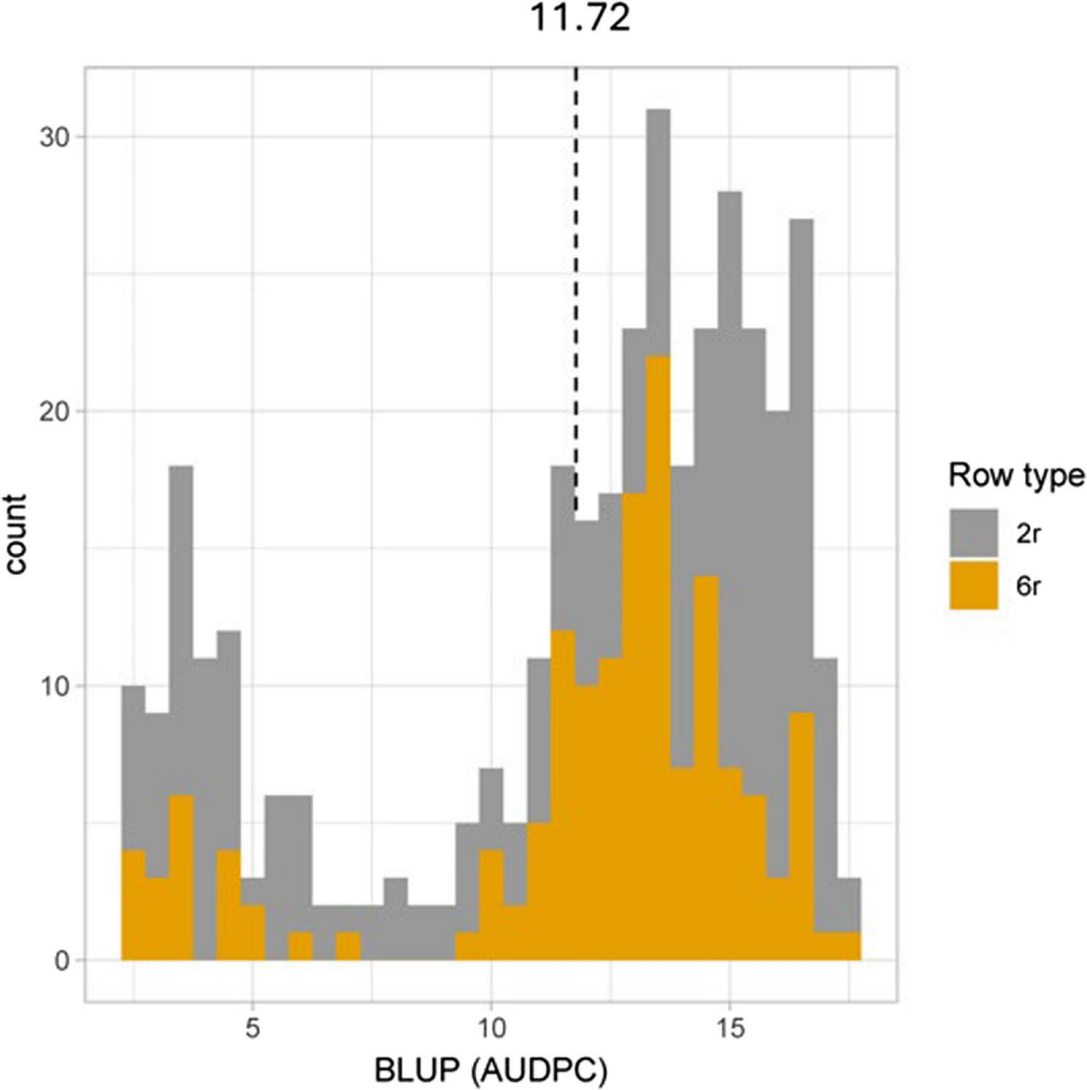
Distribution of best linear unbiased prediction (BLUP) values for powdery mildew disease among the tested spring barley breeding lines. The grey-colored bar represents the distribution of two-row barley (219 genotypes, including the check), referred to as 2r in the legend. The yellow-colored bar represents six-row barley (151 genotypes, including check), referred to as 6r in the figure. The mean BLUP (AUDPC) of the entire population was 11.72

The ANOVA revealed significant phenotypic differences in BLUP (AUDPC) values among genotypes, indicating variation in resistance to powdery mildew (Table 1). BLUP (AUDPC) values ranged from 2.43 to 17.38, and their distribution is shown in Fig. 2. The check cultivar, Judit (two-row), exhibited the highest BLUP (AUDPC) value of 17.38 while the other check cultivar, Anneli (six-row), attained 14.45. The broad-sense heritability was above 0.80 for both subpopulations. The calculated BLUP (AUDPC) values were directly used for PS by ranking the genotypes in ascending order at different selection thresholds based on their observed resistance performance (Supplementary Table S1 and S2).

**Table 1:** Analysis of variance (ANOVA) for PM resistance from 370 commercial barley breeding lines together with two commercial barley cultivars recorded from two experiments

Analysis	Population	
	two-row (2r)	six-row (6r)
Minimum BLUP	2.43	2.47
Maximum BLUP	17.29	17.38
Mean	11.47	12.1
Heritability	0.89	0.85
CV	22.98	18.59
Genotype significance	8.5E-51	1E-28

### Selection method 2: association between genetic merit and phenotypic values

The genetic merit was calculated for each genotype using the allelic effects of significant markers, for both the two-row and six-row subpopulations individually. A Pearson correlation was calculated between genetic merit and BLUP (AUDPC) values, resulting in a correlation of 0.76 for two-row and 0.73 for six-row, which indicates that a significant proportion of phenotypic variation was captured.

#### Genome-wide association analysis for powdery mildew resistance

**Fig. 3:**
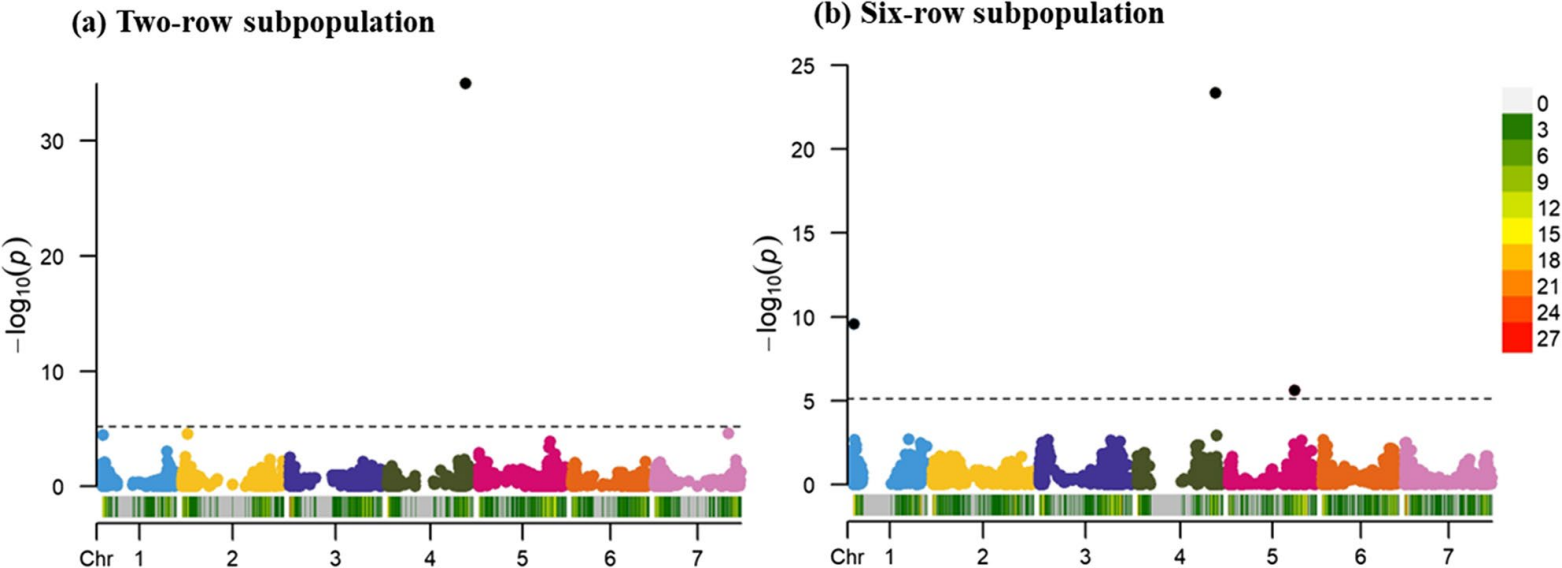
Manhattan plot showing marker-trait associations for powdery mildew resistance in the (**a**) two-row and (**b**) six-row barley subpopulations. Each point represents SNP marker plotted against its genomic position and –log₁₀(p) value. The dashed line indicates the Bonferroni significance threshold. The color-coded bar below the X-axis shows SNP density across the genome, with the legend indicating density levels from low (white) to high (red)

GWAS was conducted independently for the two-row and six-row spring barley subpopulations, as described in the Methods section, and by this, three markers (using 6,915 markers) for six-row and one marker for two-row (using 8,088 markers), significant at the Bonferroni threshold, were obtained **(**Table 2). The Manhattan plots for these two GWAS analyses are illustrated in Fig. 3.

**Table 2:** List of significant MTAs for powdery mildew resistance detected in two-row and six-row spring barley subpopulations

SNP	Chr	Pos. (Mbp)	*P* value	Effect	MAF	alleles*	PVE (%)	detected population
JHI_Hv50k_2016_4209	1	4.51	2.74E-10	-1.22	0.229	**A/**G	8.40	six-row
JHI_Hv50k_2016_266364	4	589.09	4.74E-24	-3.11	0.199	C/**T**	50.57	six-row
JHI_Hv50k_2016_321026	5	498.03	2.33E-06	-1.13	0.137	**G**/T	6.13	six-row
JHI_Hv50k_2016_266643	4	589.33	1.00E-35	-3.25	0.355	**C**/T	61.15	two-row

*Chr* Chromosome, *Pos* Position in Mega base pair (Mbp), *MAF* Minor Allele Frequency; Effect, Allelic effect (*the allele written in bold represents the favorable allele; *PVE* Phenotype Variance Explained by the detected marker (%)

The MTAs to the detected markers were in the six-row subpopulation; JHI-Hv50k-2016-4209 on Chr 1 H (at 4.51 Mbp, explaining 8.40% PVE), JHI-Hv50k-2016-266364 on Chr 4 H located at 589.09 Mbp, and this MTA exhibited a substantially high PVE of 50.57%, and JHI-Hv50k-2016-321026 on Chr 5 H presented at 498.03 Mbp, with 6.13% PVE. The single MTA identified in the two-row subpopulation, JHI-Hv50k-2016-266643, was located at the distal end of Chr 4 H (589.33 Mbp), and accounted for 61.15% PVE, which demonstrated its large effect on the powdery mildew resistance phenotype. The significant MTAs are summarized in Table 2, and in this study, the ranking and selection of genotypes for powdery mildew resistance were based on these MTAs together with their estimated allelic effects, which were later used to calculate genetic merit.

### Selection method 3 and 4: Genomic prediction Model performance

#### Marker identification and testing their consistency for GWAS-incorporated GP model

**Fig. 4:**
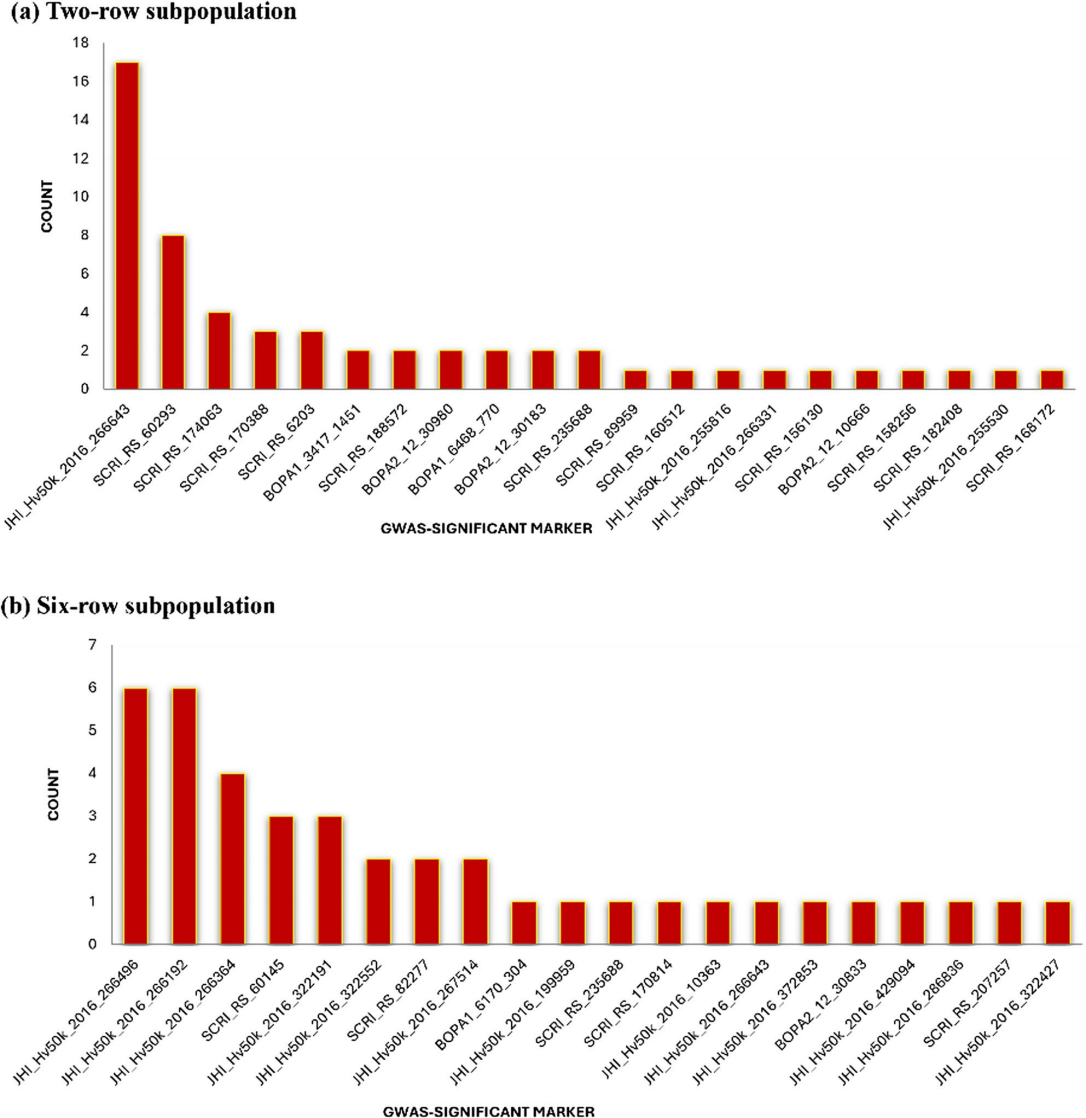
Markers selected from 20 iterations of GWAS using independent training set. **a** Number of markers detected in two-row subpopulation. **b** Number of markers detected in six-row subpopulation

A total of 39 unique MTAs were detected, and among them, two MTAs were shared between the two subpopulations. The marker JHI-Hv50k-2016-266643 was most consistently found in the two-row population, appearing in 17 out of 20 runs. SCRI_RS_60293 showed up 8 times, and SCRI_RS_174063 appears four times. Two other markers showed up three times, while the rest were detected only once. In the six-row population, JHI_Hv50k_2016_266496 and JHI_Hv50k_2016_266192 appeared in each of the six runs. JHI_Hv50k_2016_266364 was found in four runs while SCRI_RS_60145 and JHI_Hv50k_2016_321991 were in three runs. The remaining markers were observed at maximum twice (Fig. 4 and Supplementary Table S3).

#### Predictive ability across the GWAS-incorporated and standard GP models

**Fig. 5:**
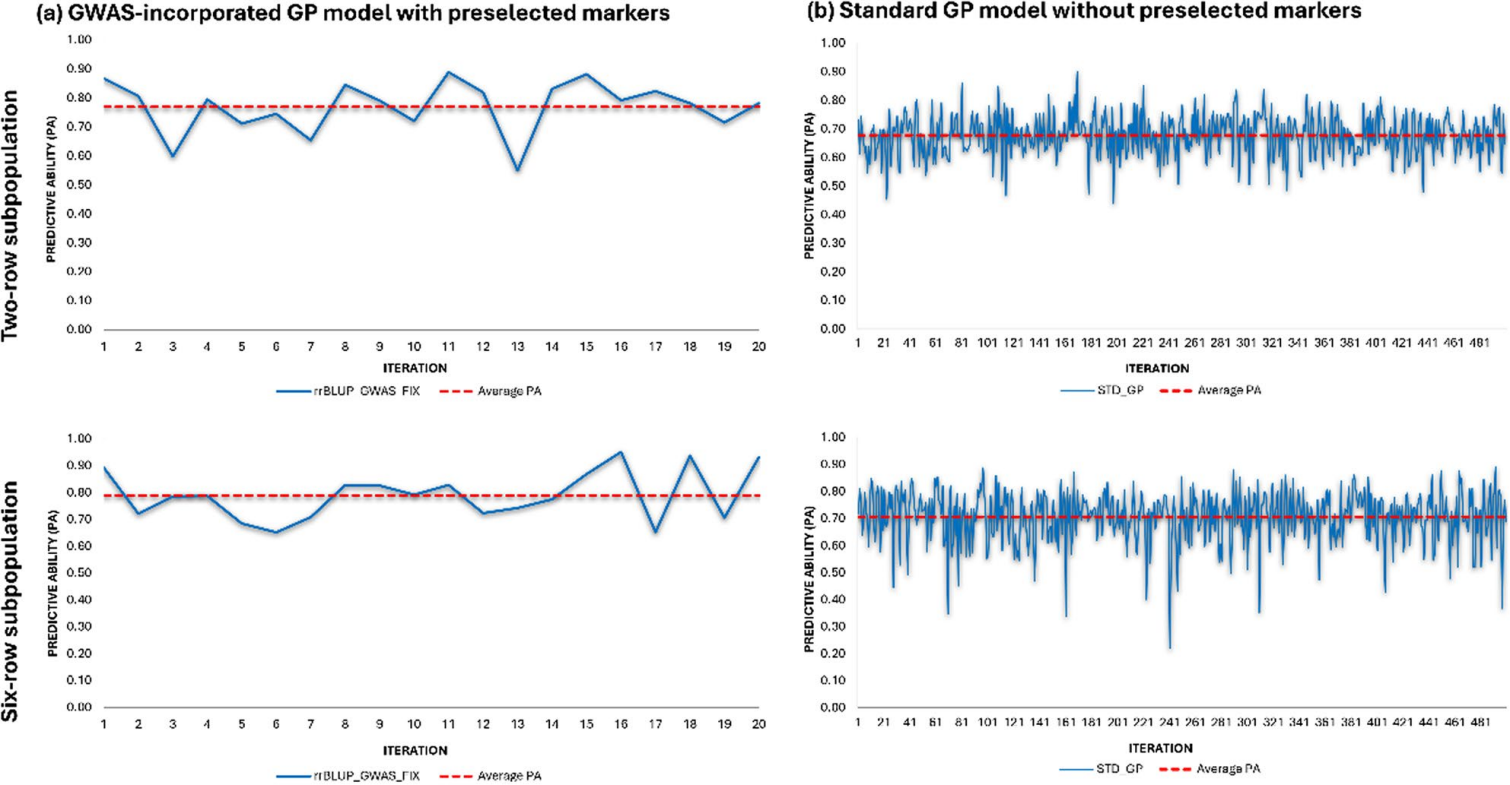
PA of two genomic prediction models in two-row (top row) and six-row (bottom row) subpopulations. The left panel (Fig. 5**a**.) represents the PA of GWAS-incorporated GP model (GWAS_FIX_rrBLUP) which included Bonferroni-significant markers as fixed effects while the remaining markers were included as random effects across 20 iterations. Right panel (Fig. 5**b**.) represents the PAs of the Standard GP model (STD_rrBLUP) across 500 iterations including all the tested markers as random effects. The yellow line represents the average PA across all iterations for each subpopulation. The average PA of two-row subpopulation were 0.68 for STD_rrBLUP and 0.77 for GWAS_FIX_rrBLUP. The average PA of six-row subpopulation were 0.71for STD_rrBLUP and 0.79 for GWAS_FIX_rrBLUP

The predictive abilities (PAs) of the tested GP models across different iterations are illustrated in Fig. 5, and the detailed results are provided in Supplementary Table S4 to S7.

The GWAS-incorporated GP (GWAS_FIX_rrBLUP) model showed a higher average PA than the standard GP (STD_rrBLUP) model in both subpopulations. For the two-row subpopulation (Fig. 5a, top left panel), the average PA was 0.77, with the PA fluctuating between 0.55 and 0.89 across 20 iterations. For the six-row subpopulation (Fig. 5a, bottom left panel), the GWAS-incorporated GP model achieved a comparable average PA of 0.79, with a range of 0.65 to 0.95.

In the two-row subpopulation, the average PA of STD_rrBLUP model was 0.68, with a range of 0.44 to 0.90 across 500 iterations (Fig. 5b, top right panel). For the six-row subpopulation, the model yielded an average PA of 0.71, with a broader range of 0.22 to 0.89 (Fig. 5b, bottom right panel). The GEBVs derived from both the Standard GP model and the GWAS-incorporated GP model were used to rank genotypes, forming the basis for genomic selection at predefined thresholds which were discussed below.

### Genotype selection patterns and consistency across different selection strategies

**Fig. 6:**
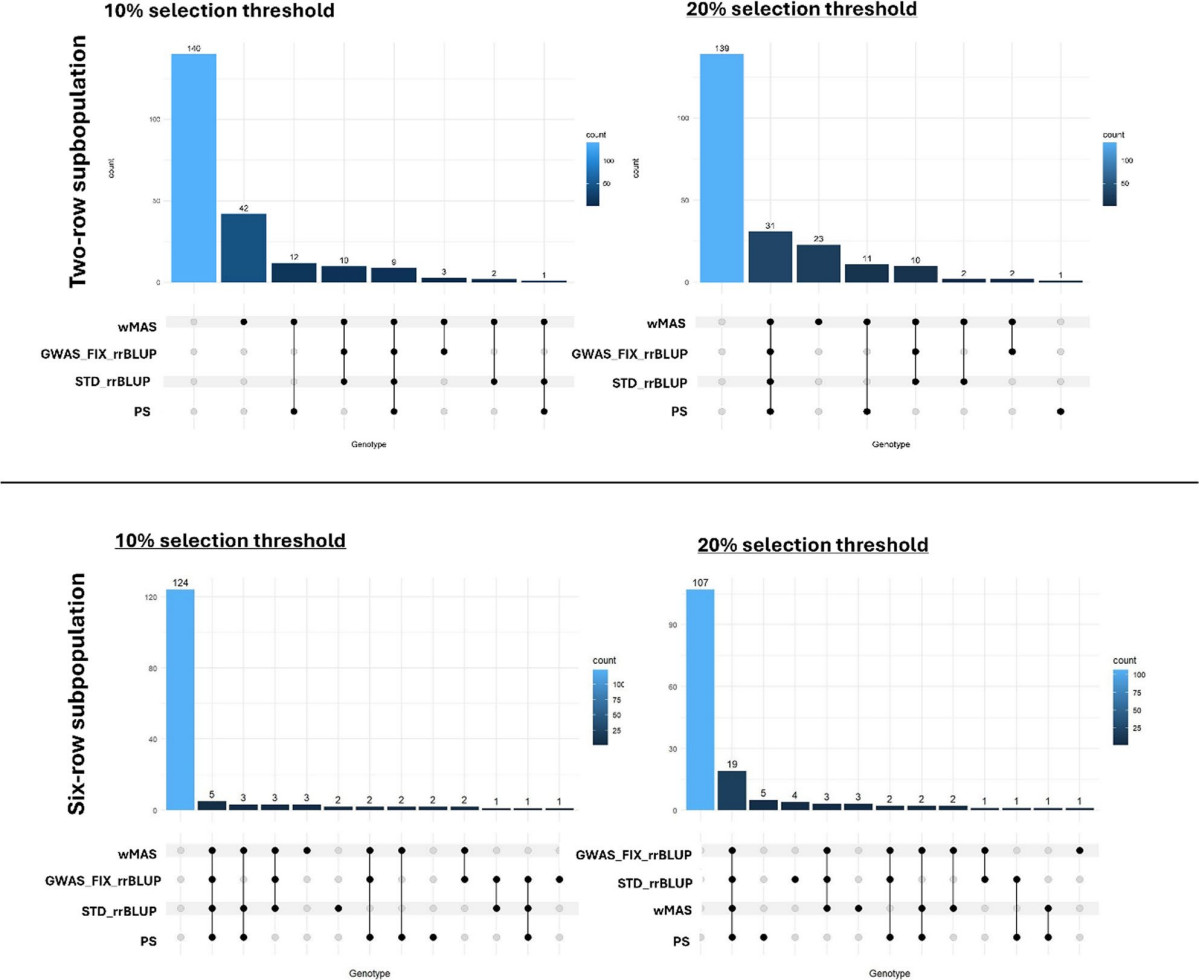
UpSet plots illustrating the genotypes selected across four selection methods (PS: phenotypic selection; wMAS: weighted marker-assisted selection; GWAS_FIX_rrBLUP: GWAS-incorporated genomic prediction; and STD_rrBLUP: standard genomic prediction) in the two-row and six-row barley subpopulations under 10% (left) and 20% (right) selection thresholds. Black circles indicate methods that selected a given genotype, whereas gray circles indicate methods that did not. Connecting lines represent genotypes selected by multiple methods, indicating shared selection options

To evaluate the consistency of the genotype selection strategies for powdery mildew resistance, two selection thresholds were applied: the top 10% and 20% most resistant genotypes in each subpopulation. Thereafter, the presence of each genotype were assessed across the four selection methods: PS, wMAS, STD_rrBLUP, and GWAS_FIX_rrBLUP. The genotype selections among these four methods are here visualized using the UpSet plots (Fig. 6), while detailed information on the genotypes and their corresponding groups according to the selection methods are provided in Supplementary Tables S8 to S11.

#### Genotypes selection in two-row subpopulation

By the use of the 10% threshold level, nine genotypes representing potential candidates for powdery mildew resistance due to both resistant phenotypes and strong genetic backgrounds were collectively chosen by all four selection methods used here (Fig. 6**)**. A total of 139 genotypes fell outside the 10% selection threshold. Nine genotypes were selected using three selection methods, specifically through two combinations: one excluding PS and the other excluding GWAS_FIX_rrBLUP. Twenty genotypes were jointly selected by two methods (eight genotypes by PS and wMAS, nine by GWAS_FIX_rrBLUP and wMAS; three by STD_rrBLUP and wMAS). Under their own selection criteria, wMAS identified 41 unique genotypes while STD_rrBLUP identified only one genotype (Fig. 6).

Under the 20% selection threshold, 23 genotypes were commonly selected by all four selection methods. Additionally, nine genotypes were consistently identified by marker-based and genomic-based selections but were not selected by PS. Ten genotypes were co-selected by three methods where PS and wMAS were always included along with either GWAS_FIX_rrBLUP or STD_rrBLUP. Sixteen genotypes were selected by two methods, with wMAS as a primary selection approach, while the second method varied among GWAS_FIX_rrBLUP, STD_rrBLUP, and PS. Eight genotypes were uniquely selected by STD_rrBLUP, while wMAS selected 21 genotypes independently, and PS selected only one genotype. All four methods consistently excluded the same 131 genotypes **(**Fig. 6**)**.

#### Genotypes selection in six-row subpopulation

At the 10% selection threshold, 124 genotypes were not selected by any method, indicating their poor performance and limited potential for breeding. In contrast, five genotypes were selected by all methods which suggests their superior phenotypic performance and genetic background for durable resistance. Nine genotypes were selected by three methods, five genotypes by two methods in different combinations, selected five genotypes. The remaining eight genotypes were uniquely selected by only one method **(**Fig. 6**)**.

At 20% threshold, the genotype selection pattern was similar to that at 10% threshold, but with more genotypes included. Nineteen genotypes were consistently selected by all methods, while the number of genotypes not selected by all methods decreased to 107. Three methods commonly selected seven genotypes, while five were identified by two methods, in different combinations. The remaining 13 genotypes were exclusively selected by only one method (Fig. 6).

## Discussion

The current study revealed substantial phenotypic variation for powdery mildew resistance in the evaluated spring barley population, with a high broad-sense heritability indicating genetic backgrounds as predominant factors. While most of the tested population was susceptible, the existing genetic diversity and heritable variation provide a great opportunity for effective breeding. The identification of MTAs and QTL offers a direct path to a wMAS approach. Our study found that a positive correlation between the genetic merit and BLUP values indicates wMAS effectively captured a large portion of phenotypic variation in both populations. However, relying solely on major genes has significant limitations. Due to their pleiotropic effects, negative trade-offs such as susceptibility to other pathogens and yield loss can be occurred [35, 36]. This indicates the need for broader approaches that are less dependence on major genes. To address this, genomic selection uses genome-wide markers reducing the reliance on single genes and contributing to more durable resistance [18]. Our GWAS results support this by identifying 39 markers forming 21 QTL of which several have been previously reported as summarized in Table 3. Detail information of these QTL are provided in Supplementary Table S3. A limitation to consider is that the virulence profile of the field-derived powdery mildew inoculum was not defined, meaning that part of the observed phenotypic variation may reflect variability within the pathogen population itself. The absence of pathogen characterization could also have contributed to differences in disease progression between experiments.

Some MTAs were consistently detected across different runs, suggesting they may represent major-effect loci for further functional analysis. In contrast, MTAs identified less frequently or even once suggest they might be false positives. However, some of them may be truly associated with powdery mildew resistance, but with small effects, or they may be specific to particular genetic backgrounds. Validation using separate or larger populations would be required to confirm their usefulness in barley breeding programs.

**Table 3:** Summary of identified QTL and their comparison to previously reported loci

Identified QTL	Position (Mbp)	Population	Previously Reported QTL/Gene	Comparison to known loci	References
QTL_1H_1	4.18–4.94	Both	*Mla* locus (~ 7 Mbp) & 30 described race-specific resistance specificities	Plausible a variant of *Mla*	[37–40]
QTL_1H_2	10.55	six-row	Known QTL (11.3 Mbp)	Same QTL	[41]
QTL_1H_3	493.32	two-row	Qrbg_1H_3 (~ 494.08 Mbp)	Same QTL	[4]
QTL_2H_1	26.54	two-row	Known QTL (29 Mbp)	Same QTL	[41]
QTL_2H_2	41.78	two-row	Known QTL (38.1 Mbp)	Same QTL	[41]
QTL_2H_3	263.47	two-row	no proximal QTL	New QTL	No prior reference
QTL_2H_4	664.04	two-row	no proximal QTL	New QTL	No prior reference
QTL_3H_1	541.7	six-row	no proximal QTL	New QTL	No prior reference
QTL_4H_1	536.71–538.09	two-row	no proximal QTL	New QTL	No prior reference
QTL_4H_2	570.52	two-row	no proximal QTL	New QTL	No prior reference
QTL_4H_3	586.89–590.48	Both	*mlo* locus (~ 589.3 Mbp)	Same QTL	[42]
QTL_5H_1	1.32–2.22	two-row	Qhv_PM-5 H.1 (2.54 Mbp)	Same QTL	[43]
QTL_5H_2	25.89	six-row	no proximal QTL	New QTL	No prior reference
QTL_5H_3 to QTL_5H_6	~ 500–550	Both	*Mlj* locus (imprecise) & four different QTL	Potentially corresponds to *Mlj*	[41, 44]
QTL_6H_1 & QTL_6H_2	9.84, 37.02	six-row	*Mlh* locus (imprecise)	Potentially corresponds to *Mlh*	[6, 44]
QTL_6H_3	554.11	six-row	no proximal QTL	New QTL	No prior reference
QTL_7H_1	557.5	two-row	no proximal QTL	New QTL	No prior reference

The results showed the role of known major genes in powdery mildew resistance. Among the three QTL identified on chromosome 1 H, QTL_1H_1 is proximal to the well-known *Mla* locus suggesting it may represent a resistant variant within this major gene [37–40]. Additionally, QTL_1H_3 overlapped with a previously identified QTL, Qrbg_1H_3 and this QTL is reported to be associated with several plant defense-related proteins and enzymes [45–48]. Furthermore, two QTL detected on chromosome 2 H and most QTL on chromosome 5 H aligned with previously reported loci associated with seedling-stage resistance. The presence of the durable *mlo* gene on chromosome 4 H was also confirmed and it overlapped with QTL_4H_3 from our study. Most of the top-selected genotypes carried alleles closely linked to this region, suggesting that *mlo*-mediated resistance is present in the evaluated panel. However, since the causal *mlo* allele was not directly genotyped in this study, its presence cannot be confirmed for all individuals, and balancing *mlo*-based powdery mildew resistance with potential agronomic trade-offs remains important. While the precise position of the known *Mlh* locus is not available, though it is generally found near the short arm of chromosome 6 H. QTL_6H_1 and QTL_6H_2 from the current study may correspond to it since they were also proximal to this region [6, 44]. In addition to validating known loci, our study identified eight new QTL associated with powdery mildew resistance. These newly detected loci were located on chromosomes 2 H, 3 H, 4 H, 5 H, 6 H, and 7 H, consistent with the positions detailed in Table 3. Notably, some QTL such as QTL_3H_1 and QTL_7H_1, colocalized with QTL previously reported as adult-plant resistance. This suggests that these QTL are valuable for broadening resistance spectrum throughout the plant’s life cycle. Validation of these novel QTL will require testing in biparental or multi-parent populations, which was beyond the scope of the current study; however, such evaluation remains important for future validation efforts.

Previous studies have shown different results, some reported that including specific markers as fixed effects in genomic prediction models improved prediction accuracy while others found no effect or reduced accuracy [13, 16, 49–52]. In the present study, performance ranking among the genomic selection methods resulted in that the GWAS-incorporated GP model consistently achieved the highest PA, followed by wMAS, with the Standard GP model having the lowest PA. The superior performance of the GWAS-incorporated GP model is due to its effective use of GWAS-significant markers and that it optimizes the prediction [50]. The better outperformance of wMAS compared to the Standard GP model reflects the dominance of major-effect markers on powdery mildew resistance in these subpopulations. In the Standard GP model, all markers are uniformly shrunk toward zero, which reduces the effects of major loci and weakens predictions when dominant genes are present [34, 50].

The frequently detected MTAs observed in both subpopulations highlights the presence of a consistent genetic association with powdery mildew resistance in the full set of genotypes. However, MTAs detected only in random subsets of genotypes rather than the full subpopulation are likely population-specific and can be overlooked in broader analyses. Apart from these marker results, the different genetic architecture between the two subpopulations also explains the overall performance of the genomic prediction models. The six-row subpopulation outperformed the two-row subpopulation in both genomic prediction models, and this is likely due to the presence of both major genes and a favorable polygenic background. The ability of genomic prediction models to capture the cumulative effect of small-effect loci and the major genes, contributes to a high overall predictive accuracy [10, 52]. Additionally, wMAS was also notably effective in both subpopulations, showing a high correlation between genetic merit and BLUP values. However, genomic prediction appears more suitable as it has a more complex genetic architecture captured by genome-wide prediction models whereas resistance in the two-row subpopulation is mainly controlled by major-effect markers.

In addition to the different genetic architecture of the two subpopulations, the genotype selection patterns also indicated a fundamental difference. In the two-row subpopulation, a large group of genotypes were exclusively selected by wMAS revealing the dominance of major-effect QTL. This exclusive selection highlights that these genotypes have an unfavorable polygenic background and therefore they were not selected by the other methods [53]. In contrast, the six-row subpopulation exhibited a more diverse genetic architecture. The wMAS-exclusive group was smaller compared to the two-row subpopulation which suggests a strong polygenic background in the six-row subpopulation. These architectural differences were further reflected in how genotypes were selected under different thresholds.

When the selection threshold was relaxed, both the number of selected genotypes and the overlap among methods increased. Additionally, it allowed borderline genotypes with different combinations of major and minor effects, or strong phenotypic performance, to be included by multiple selection methods. Analysis of selection patterns between phenotypic and genetic approaches showed that genetic analyses can detect hidden signals that may be masked or only partly expressed during phenotypic evaluation. The differences between phenotypic and genomic selections may be due to environmental variation. This includes factors like temperature, light distribution, moisture, and humidity [54, 55].

The contrasts were not only driven by environmental variation but also by the different assumptions and algorithms underlying each selection method. For example, the GWAS-incorporated GP model can reduce its flexibility by explicitly fixing major-effect markers without shrinkage. As a result, the model may overfit and fail to predict genotypes that depend more on polygenic backgrounds [52, 56, 57]. In addition, some genotypes were consistently identified by phenotypic selection, wMAS, and the GWAS-incorporated GP, but not by the Standard GP. This shows how the Standard GP model underestimates genotypes with major-effect alleles since it shrinks all marker effects toward zero [34, 50].

These differences in selection outcomes are important when considering breeding strategy. In the two-row subpopulation, wMAS could be useful to rapidly select genotypes that carry the major-effect QTL. However, to develop more robust and durable resistance, a whole-genome approach is necessary to select genotypes with a superior polygenic background. In the six-row subpopulation, resistance comes from diverse resistance mechanism and therefore genomic selection is a more suitable option for long-term improvement than relying on a single dominant locus.

## Conclusions

This study suggests that incorporating GWAS-identified markers into genomic prediction models can improve prediction accuracy for powdery mildew resistance in spring barley. The identified QTL from this study can be validated across multiple locations and also can be incorporated into breeding pipelines to develop powdery mildew resistant cultivars. Since these QTL were detected at the seedling stage, validating their effects at the adult-plant stage could be done as future work. Furthermore, developing multi-trait genomic prediction models that include powdery mildew resistance together with traits such as yield could provide breeders with a more efficient selection strategy.

## Supplementary Information

Supplementary Material 1.

Supplementary Material 2.

## Data Availability

The phenotypic data sets, GWAS results and genomic prediction results that support the findings of this study are provided in additional files. All significant SNPs associated with powdery mildew resistance are included in the Supplementary Materials (Supplementary Table S3). The genotypic datasets analyzed during the current study are not publicly available due to intellectual-property restrictions owned by Lantmännen, our industry partner, but are available from the corresponding author on reasonable request.

## Abbreviations

QTL: Quantitative Trait Loci
AUDPC: area under disease progress curve
MTAs: marker-trait associations
GWAS: genome-wide association study
GP: genomic prediction
PA: predictive ability
GEBV: genomic estimated breeding values
PS: phenotypic selection
wMAS: weighted marker-assisted selection
GS: genomic selection
DPI: days post inoculation
FDR: False discovery rate
MAF: minor allele frequency
LD: linkage disequilibrium
BLUP: best linear unbiased prediction
GWAS_FIX_rrBLUP: GWAS-incorporated GP
STD_rrBLUP: Standard GP
