# Supplementary material for: Comparing wMAS, GWAS, and genomic prediction for selecting powdery mildew-resistant spring barley genotypes
Source: BMC Genomics. 2025 Dec 5;26:1091. doi: 10.1186/s12864-025-12395-y (PMC12690920; doi:10.1186/s12864-025-12395-y)
Supplement: Supplementary file 2 — Supplementary Material 2. [file 12864_2025_12395_MOESM2_ESM.docx]

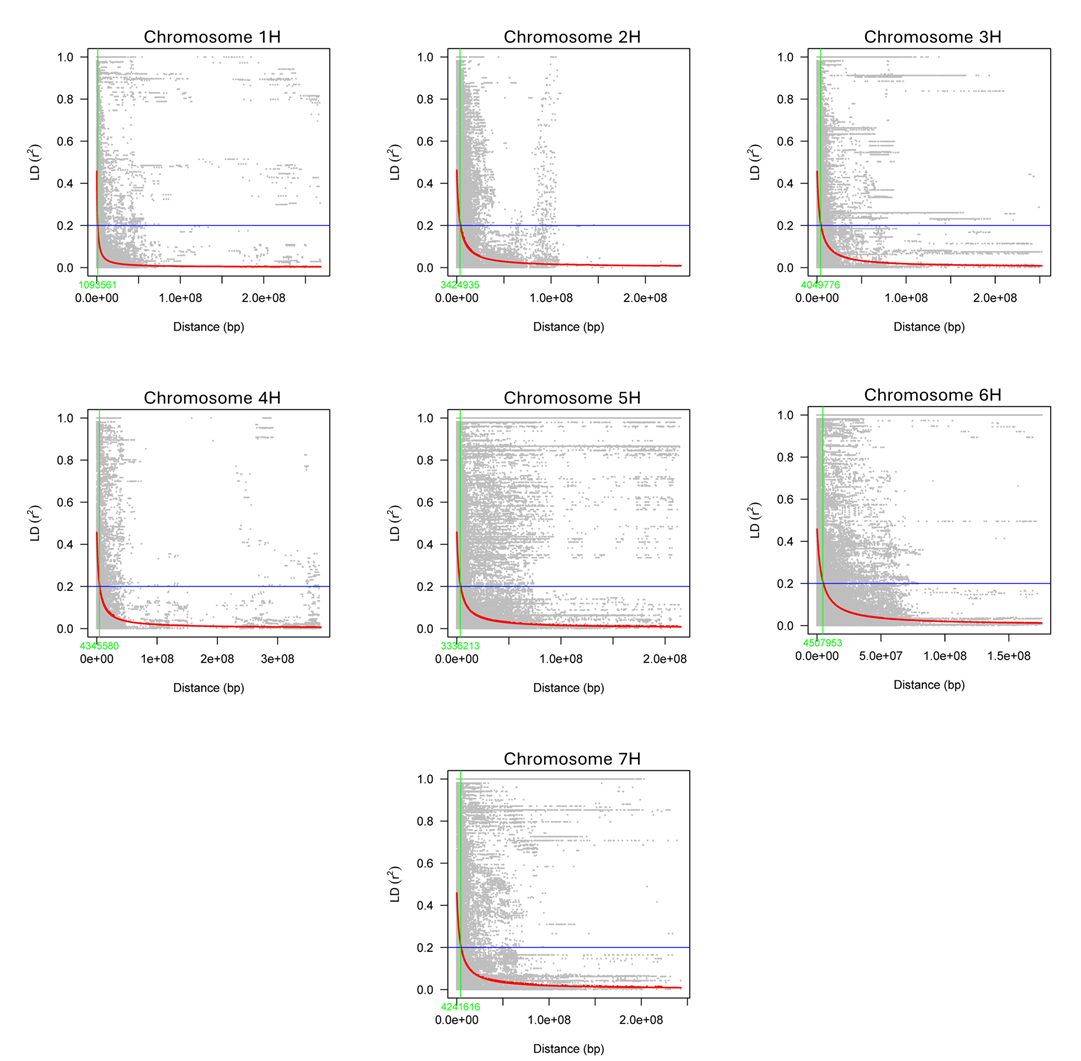


**Supplementary Figure S1.** Chromosome-wide LD decay plots fitted against the physical distance (i.e., base pairs) in 2-row subpopulation. The blue horizontal line represents the half decay distance (r2). The red curved line represents the smooth regression model fitted to LD decay. The green vertical line represents the distance between the marker pairs which intersects the half decay and the LD decay curve.

**
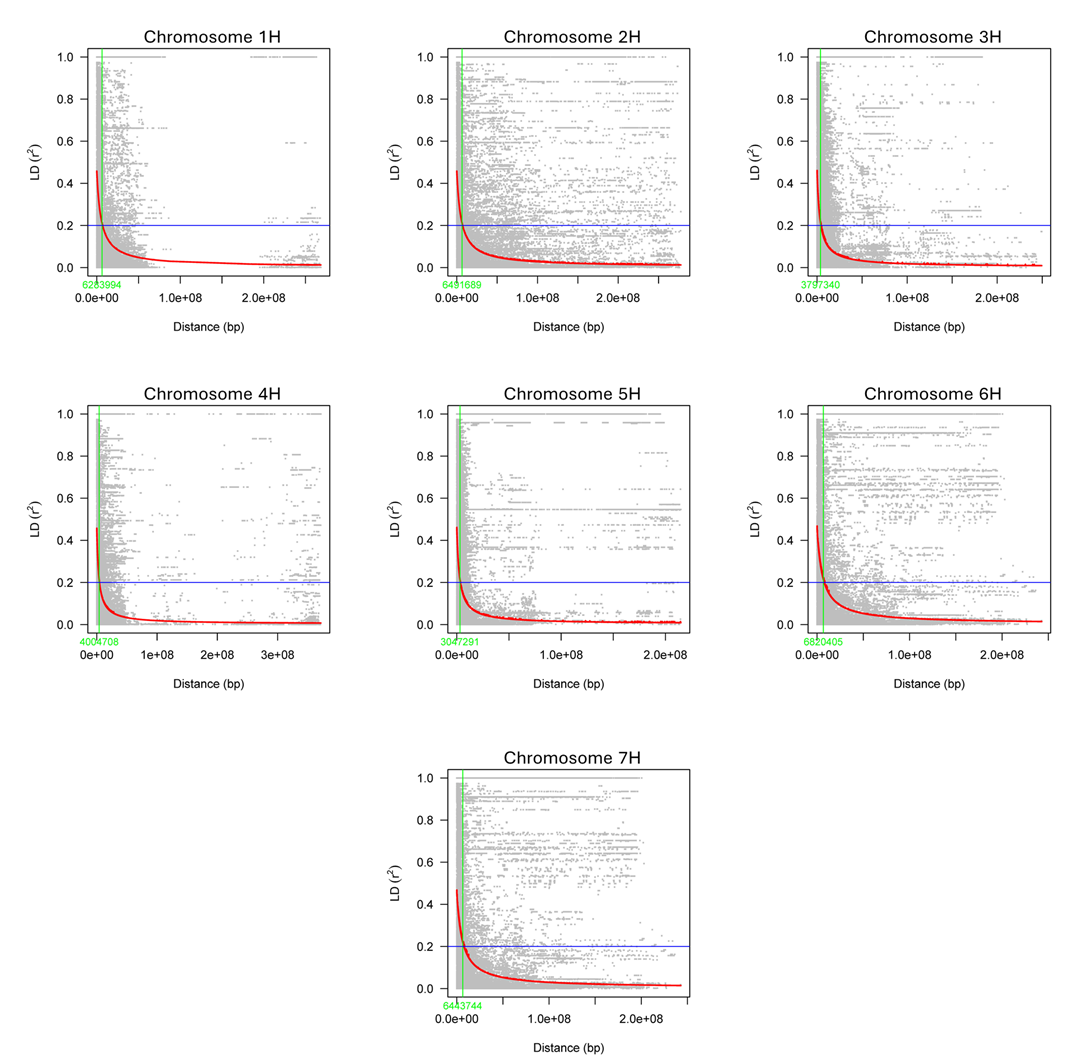
**

**Supplementary Figure S2.** Chromosome-wide LD decay plots fitted against physical distance (i.e., base pairs) in 6-row subpopulation. The blue horizontal line represents the half decay distance (r2). The red curved line represents the smooth regression model fitted to LD decay. The green vertical line represents the distance between the marker pairs which intersects the half decay and the LD decay curve.
